# Anomalous Coronary Artery Origin in a Young Patient with Marfan Syndrome

**DOI:** 10.1155/2017/3861923

**Published:** 2017-12-21

**Authors:** S. B. C. P. Duarte, D. O. Beraldo, L. A. M. Cesar, A. P. Mansur, J. Y. Takada

**Affiliations:** ^1^Heart Institute (InCor), University of São Paulo Medical School, Enéas de Carvalho de Aguiar Avenue 44, 05403-000 São Paulo, SP, Brazil; ^2^Hospital Renascentista, Salvador dos Santos Nora Avenue 76, 37550-000 Pouso Alegre, MG, Brazil

## Abstract

Marfan syndrome is an autosomal dominant genetic disorder that affects connective tissue and is caused by mutations in the fibrillin 1 gene present at chromosome 15. Aortic aneurysm is its main complication, and along the dilation of the aorta root and its descending portion (60–100%), with secondary aortic insufficiency, it increases risk of acute aortic dissection and death. Coronary artery anomalies affect between 0.3% and 1.6% of the general population and are the second leading cause of sudden death in young adults, especially if the anomalous coronary passes through aorta and pulmonary artery. The anomalous origin of the left main coronary artery in the right Valsalva sinus has a prevalence of 0.02%–0.05% and is commonly related to other congenital cardiac anomalies, such as transposition of great vessels, coronary fistulas, bicuspid aortic valve, and tetralogy of Fallot. Its association with Marfan syndrome is not known, and there is no previous report in the literature. We describe here a case of a female with Marfan syndrome diagnosed with symptomatic anomalous origin of the left coronary artery in the right Valsalva sinus.

## 1. Introduction

Marfan syndrome (MFS) is an autosomal dominant genetic disorder that affects connective tissue and is caused by mutations in the fibrillin 1 gene present at chromosome 15. It compromises mainly skeletal, cardiovascular, and ocular systems, besides skin, central nervous system, and lungs [1–3]. Clinical presentation is characterized by higher height, disproportion between upper and lower limbs (dolichostenomelia), anterior thoracic deformity (pectus excavatum or carinatum), aortic arch dilatation, aortic insufficiency, mitral valve prolapse with associated regurgitation, and many other anomalies [1–4]. Diagnosis is made by the presence of family history and evaluation of the characteristic manifestations through the “Revised Ghent-2 Nosology” [5, 6]. The treatment is based on a multidisciplinary approach, with an emphasis on cardiovascular complications.

Coronary artery anomalies (CAAs) affect between 0.3% and 1.6% of the general population [7] and are the second leading cause of sudden death in young adults, especially if the anomalous coronary origins between the aorta and the pulmonary artery [8]. There are several types of CAAs, which may be asymptomatic or manifest through myocardial ischemia, such as chest pain, dyspnea, and arrhythmias, especially to the efforts [8]. The anomalous origin of the left main coronary artery in the right Valsalva sinus has a prevalence of 0.02% to 0.05% [9] and is commonly related to other congenital cardiac anomalies, such as transposition of large vessels, coronary fistulas, aortic valve bicuspid, and tetralogy of Fallot [10]. There is no previous report in the literature about its association with Marfan syndrome. We describe here a case of a female with Marfan syndrome, who was diagnosed with symptomatic anomalous origin of the left coronary artery in the right Valsalva sinus.

## 2. Case Report

**Figure 1:**
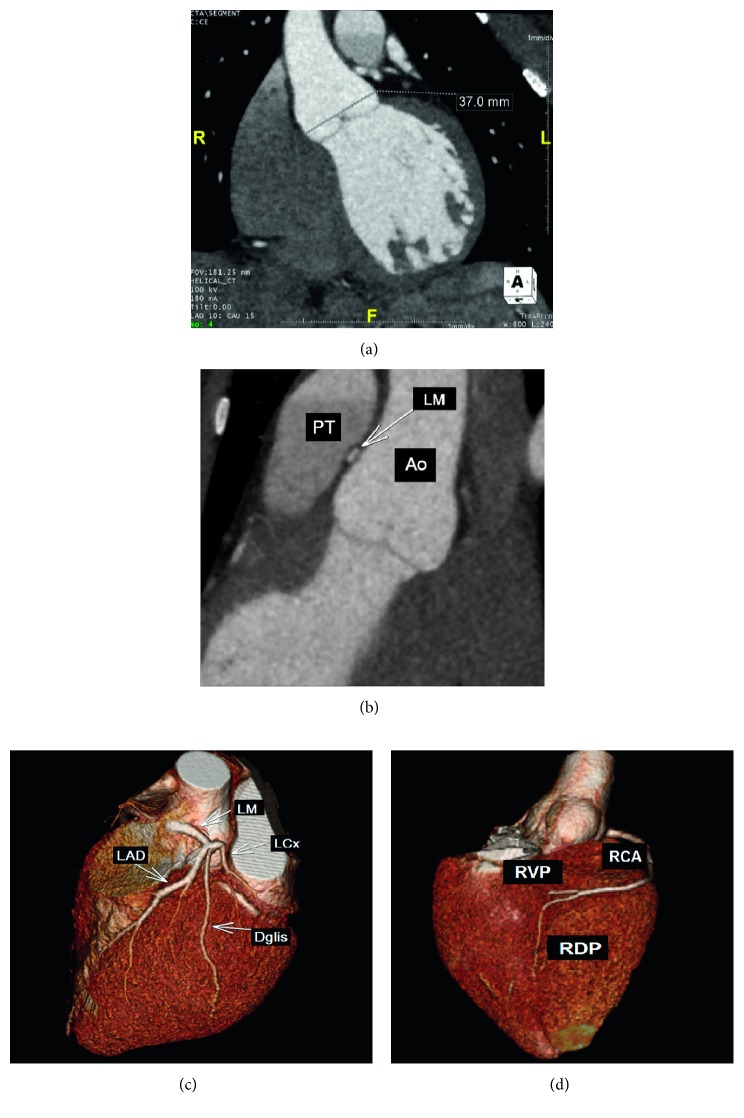
(a) CT coronary angiography demonstrates normal aortic root (37 mm) and (b, c, and d) anomalous origin of the left main coronary artery from the right coronary Valsalva sinus with a pathway between the pulmonary artery and the aorta (white arrow). LM, left main coronary artery; LAD, left anterior descending; LCx, left circumflex artery; RCA, right coronary artery; RPD, right posterior descending artery; RVP, right posterior ventricular artery; DGLIS, diagonal branch; PT, pulmonary trunk; Ao, aorta.

**Figure 2:**
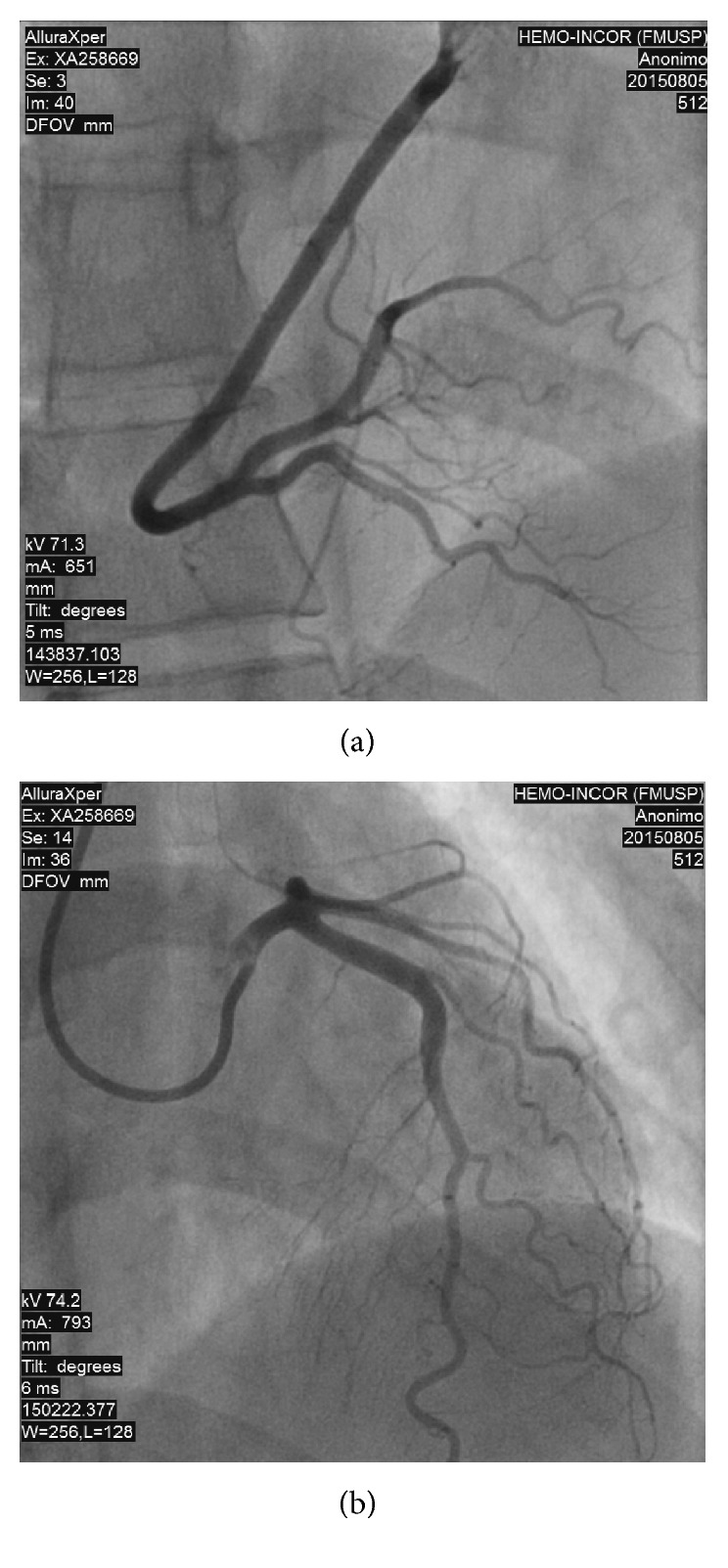
(a, b) Coronary angiography without obstructive atherosclerotic plaques.

**Figure 3:**
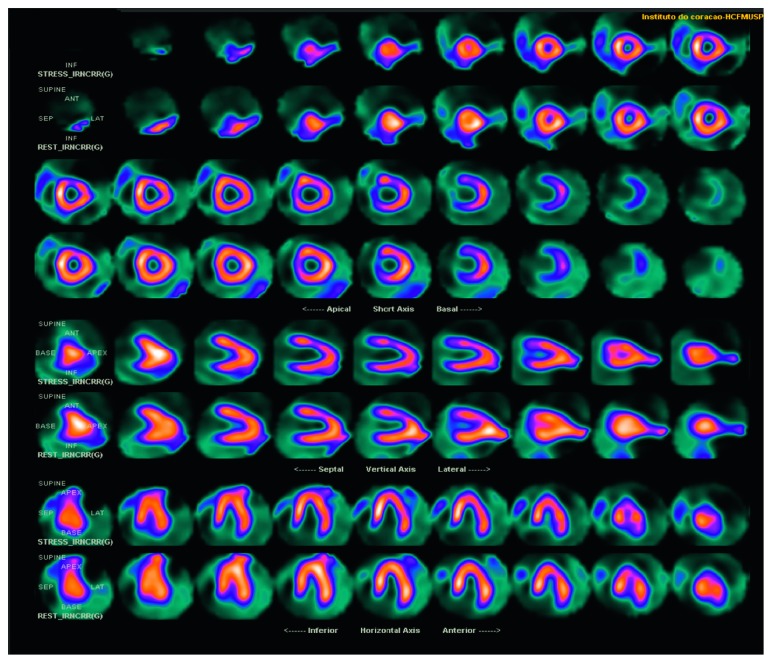
Sesta-MIBI perfusion scintigraphy showing discrete anterior wall perfusion defect (apical segment) and transient perfusion defect in the mild and basal segments of the anterior wall and in the middle segment of the anterolateral wall.

A 22-year-old female, Jehovah's witness, presented with frequent palpitations and atypical chest pain at rest and during exertion for seven years, associated with respiratory distress at moderate exertion and sporadic lower limb edema, without other symptoms. She was given atenolol 50 mg once daily as a result of palpitations, and she was a carrier of Marfan syndrome, diagnosed in childhood, without reports of previous complications related to the disease. The diagnostic criteria of Marfan syndrome, based on revised Ghent-2 nosology, was ectopia lentis and systemic score ≥ 7 points in a patient with positive family history. No history of smoking, alcohol use, or illicit drug use. She presented with typical Marfan syndrome alterations in ectoscopy (pectus carinatum deformity, hindfoot deformity, wrist and thumb signs, scoliosis or thoracolumbar kyphosis, dolichocephaly, enophthalmos, downslanting palpebral fissures, malar hypoplasia, and skin striae), and examination of the cardiovascular system was completely normal. Biochemical analysis was performed (hemoglobin: 15.3 g/dL, leukocytes: 6.510 cells/mm^3^, platelets: 245.000 cells/mm^3^, creatinine: 0.74 mg/dL, urea: 19 mg/dL, total cholesterol: 239 mg/dL (HDL: 88 mg/dL and LDL: 125 mg/dL), triglycerides: 131 mg/dL, and fasting glucose: 82 mg/dL). Electrocardiogram was normal. Exercise stress testing by the Ellestad protocol resulted in one paired ventricular premature ventricular beats, a long period of ventricular bigeminism, and absence of significant ST segment changes. The 24-hour Holter demonstrated sinus rhythm, 5.605 ventricular premature beats (<6%) with frequent bigeminism, and 16 supraventricular (<1%) premature beats, with no other changes. Echocardiogram and magnetic resonance of the heart depicted mitral valve prolapse with mild regurgitation and no other changes. Computed Tomography (CT) coronary angiography revealed the left main coronary artery with anomalous origin from the right coronary Valsalva sinus and an initial pathway between the aorta and pulmonary arteries, where there was significant luminal reduction (close to 50%) at rest, absence of atherosclerosis or luminal reduction in the other coronary arteries, and total calcium score of zero (Figure 1). After meticulous CT coronary angiography analysis, it was not possible to define intramural coronary pathway existence and there was no intravascular ultrasonography available to improve diagnostic accuracy. Coronary angiography confirmed an anomalous origin of the left coronary artery, with exit from the right coronary sinus without obstructive lesions (Figure 2). Sesta-MIBI perfusion scintigraphy demonstrated left ventricular ejection fraction of 61% at rest, and 57% poststress, discrete anterior wall perfusion defect (apical segment), and mild transient perfusion defect in the middle and basal segments of the anterior wall and the segment of the anterolateral wall (Figure 3). No aortic dilation was observed in CT aortic angiography.

After a correlation between symptoms and the presence of myocardial ischemia, the Heart Team approach decided for elective surgical treatment. Although the patient was young and did not have atherosclerotic coronary disease, the surgical group preferred to make coronary arterial bypass grafting (left internal mammary artery to the left anterior descending artery) instead of coronary unroofing or reimplantation because of the service experience and undefinition of intramural pathway existence. The procedure occurred without complications, and the patient has been followed-up asymptomatically for 6 months.

## 3. Discussion

Aortic aneurysm is the main complication of Marfan syndrome. Dilation of the aorta root and its descending portion (60–100%), with secondary aortic insufficiency, increases risk of acute aortic dissection and death. Aburawi and O'Sullivan described that aortic root dilatation develops early in MFS and was present in 35% by the age of 5 years, 68% by 19 years, and at least 80% by 40 years [11]. Other cardiac complications are less common in Marfan syndrome [12–20].

Coronary artery anomalies, although present in other syndromic pathologies, are not described in Marfan syndrome. As discussed above, its importance is related to the risk of sudden death and to events related to myocardial ischemia when the anomalous coronary pathway goes between the aorta and the main pulmonary artery, mainly if it is intramural. In addition to the compressive effect of the great vessels due to their anatomical changes by the effort, other mechanisms precipitating ischemia seem to be involved, such as intramural pathway, angulation in the artery pathway of anomalous implantation, endothelial lesion with consequent vasospasm, and higher incidence of atherosclerotic disease in this type of vessel [9, 21–25].

The anomalous origin of the left main coronary artery in the right Valsalva sinus can be classified into 4 types, based on its path: (a) pathway between the aorta and the pulmonary trunk, (b) pathway anterior to the exit of the right ventricle, (c) pathway through the supraventricular intramyocardial or subendocardial ridge, and (d) posterior pathway to the aortic root [23].

The treatment can be medical or surgical, and it depends on the age, existence of associated coronary artery disease, presence of clinical signs suggestive of myocardial ischemia and/or positive ischemic evaluation tests, presence of intramural coronary segment, and the experience of the service [6]. According to the 2008 American Heart Association/American College of Cardiology guidelines, surgical treatment is indicated in patients with any of the following: anomalous left main coronary artery coursing between the great arteries and documented coronary ischemia secondary to coronary artery compression when the artery courses between the aorta and the pulmonary artery or intramurally [26]. In regard to the ischemic tests, their sensitivity for the prediction of sudden death is unknown. Moreover, the intense physical exertion that results in sudden cardiac death is not achieved with these tests, so its hemodynamic effects are difficult to analyse. The intravascular ultrasonography has been used successfully to evaluate the dynamic aspects of the anomalous coronary intramural segment, which may help to decide the ideal treatment option [24, 26, 27]. There are three main modalities of surgical treatment, and the best choice depends on the patient's characteristics. Coronary unroofing is indicated when there is intramural coronary segment. Coronary arterial bypass grafting is indicated mainly in older patients, who have no longer life expectancies than their bypass grafts, and those with coronary atherosclerotic artery disease. Reimplantation involves transferring the coronary ostium to the appropriate sinus, but forming coronary buttons and reimplanting at an appropriate angle can be technically challenging [6, 28–30]. In our case report, there was no intravascular ultrasonography available, and the Heart Team decided on coronary arterial bypass grafting (left internal mammary artery to the left anterior descending artery). It occurred because of the service experience and undefinition of intramural pathway existence.

The case reported here describes a patient with MFS and anomalous coronary artery origin who developed clinical and laboratorial signs of coronary ischemia. The approach of these patients is very controversial; however, due to the possibility of sudden death related to the efforts, among other cardiac complications, surgical treatment was indicated. So, this rare condition of coronary anomaly is possible and must be considered in association with Marfan syndrome as described here.
